# Integrating AI into Dentistry: Technical Pathways and Clinical Applications in Oral Diseases–A Comprehensive Overview

**DOI:** 10.1016/j.identj.2025.109336

**Published:** 2026-01-14

**Authors:** Ying Yuan, Hao Zhou, Bo Wang, Chengrun Li, Baijuan Gong, Zhimin Li

**Affiliations:** School and Hospital of Stomatology, China Medical University, Shenyang 110002, China

**Keywords:** Artificial intelligence, Deep learning, Dentistry, Oral diseases, Patient care

## Abstract

The application of artificial intelligence (AI) technologies in the management of oral diseases is rapidly increasing, encompassing etiopathogenesis, disease course, diagnosis, treatment plans, and postoperative care. AI demonstrates exceptional performance in enhancing diagnostic accuracy and supporting clinical decision-making. This review provides a comprehensive overview of AI in dentistry, elucidating the neural network architectures and major implementation methods of AI. It focuses on specific application scenarios of AI-based models and evaluates their performance, and additionally highlights the limitations and promising future directions, providing insights to advance the clinical applications of AI in dentistry.

## Introduction

Artificial intelligence (AI), a field of computer science, is extensively studied and applied in various fields. The integration of AI in healthcare has revolutionized the medical field, transforming both research and clinical practice.^1^ AI technologies promote innovations in dentistry and help provide more accurate and efficient dental services, benefiting patients and dentalcare professionals.

**Fig. 1:**
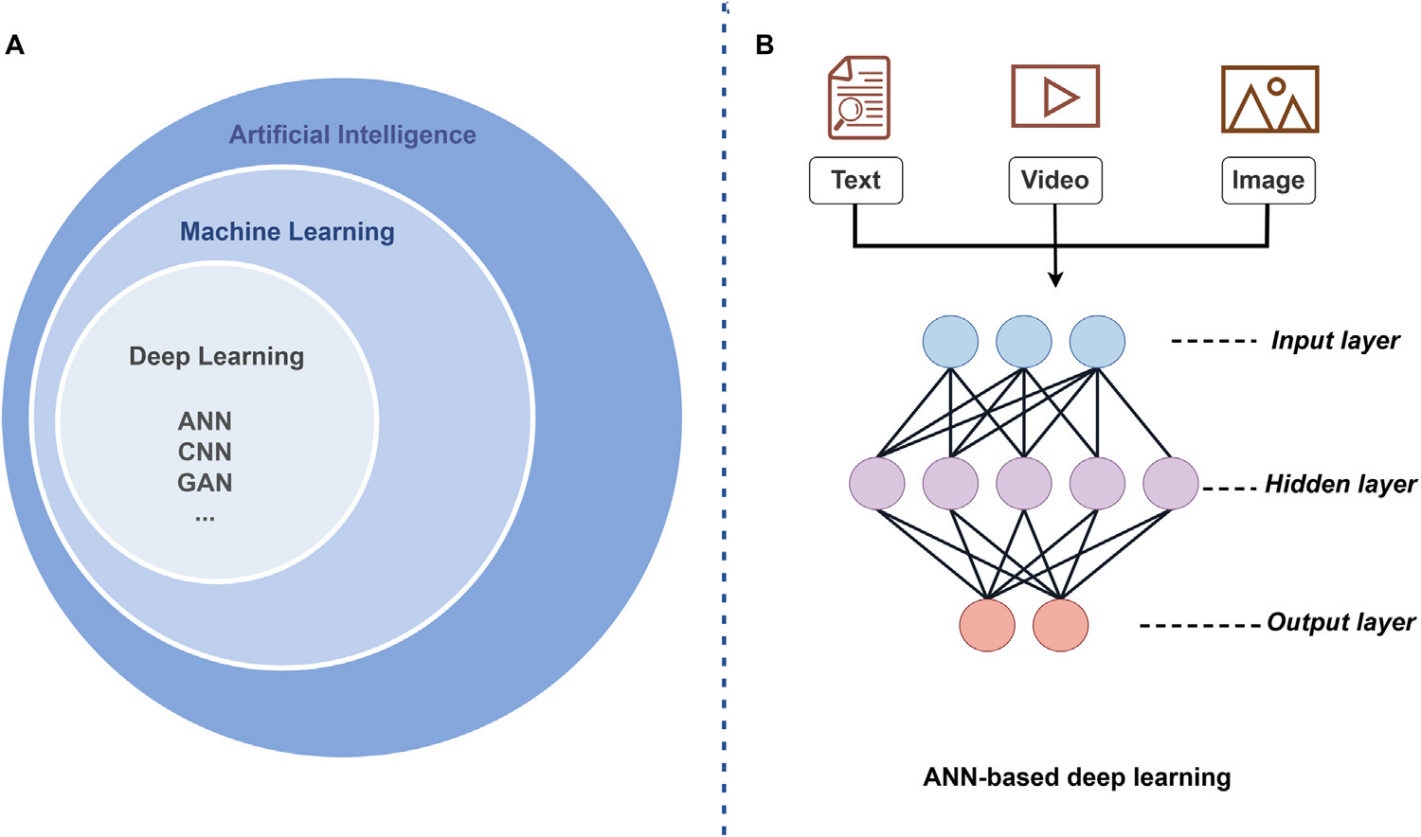
(A) AI subsets (B) ANN architecture comprising 3 core layers: input, hidden, and output. The main types of input data in medical applications include text, videos, and images.

The rapid integration of AI in dentistry mirrors its own evolutionary trajectory through key phases. Machine learning (ML), a core component of AI (Figure 1A), uses algorithms and models to allow computers to learn and identify patterns and rules by analyzing large amounts of data and predict or make decisions on new data without explicit programming.^2^ Artificial neural network (ANN) is the most popular ML model. Based on biological neurons,^3^ ANN comprises three layers: input, hidden, and output (Figure 1B). The “neurons” in each layer are the basic processing units, and interconnected “neurons” form a network that enables information processing. Deep learning (DL) uses the core structure of ANN; however, it has an increased number of hidden layers. Theoretically, DL is a subset of ML that performs complex tasks that ML cannot perform, such as natural language processing (NLP),^4^ behavior recognition,^5^ and medical image analysis,^6^ through a multi-layered neural network. The commonly used medicine-related DL algorithms include convolutional neural networks (CNN), recurrent neural networks (RNN) and several extended models such as generative adversarial networks (GAN), multimodality models (MM), reinforcement learning (RL), and transformers.^7^

In dentistry, input data of the neural network include image (lesion photographs, computed tomography [CT] images, and pathology images) and textual (electronic medical records [EMR]) or audio (consultations and case discussions) data. These data streams are transformed into clinically actionable outputs via the DL architecture that ultimately help clinicians: diagnostic results, clinical treatment planning, disease prediction or prognosis, and patient management.^8^^,^^9^

This review focuses on the technical implementation pathways and mechanisms of AI in dentistry, the current status of AI applications, and the potential risks and challenges. The performance of AI models in cross-disciplinary integration varies considerably, and is influenced by model architecture, scale and quality of input data, and diversity of performance metrics, highlighting the critical need to evaluate and compare the effectiveness of diverse AI methodologies. This review elucidates the ‌standards for performance evaluation and conducts a horizontal comparison of AI models across different studies using a unified assessment framework, encompassing metrics such as accuracy, sensitivity, and specificity, simultaneously identifying the respective limitations.

This review further aims to provide insights for the adoption of AI-driven solutions in oral healthcare by systematically analyzing the transformation pathways of AI in oral disease diagnosis and treatment and critically evaluating recent research outcomes, thereby promoting the technological evolution of dentistry.

## Methods

Relevant studies on AI in dentistry were retrieved from PubMed and Web of Science using the following MeSH terms and keywords along with their synonyms, near-synonyms, and abbreviated forms to ensure comprehensive coverage: “artificial intelligence,” “deep learning,” “neural network,” “automatic detection,” “natural language processing,” “CBCT,” “caries,” “periapical lesion,” “periodontitis,” “temporomandibular disorders,” “oral cancer,” “OSCC,” and “dental crown.” Inclusion criteria were as follows: peer-reviewed articles and highly cited systematic reviews on AI applications in dentistry published in the last 5 years (2020-2025), except for seminal works. Exclusion criteria were as follows: duplicate entries and studies with unclear methodologies or questionable data quality. Additionally, reference lists of selected studies were hand-searched to identify relevant literature. A literature management tool was used for efficient categorization and organization of the search results, ensuring methodological transparency, minimizing selection bias, and providing robust evidence for study conclusions.

## AI in dentistry pathways

### AI image recognition technology

**Fig. 2:**
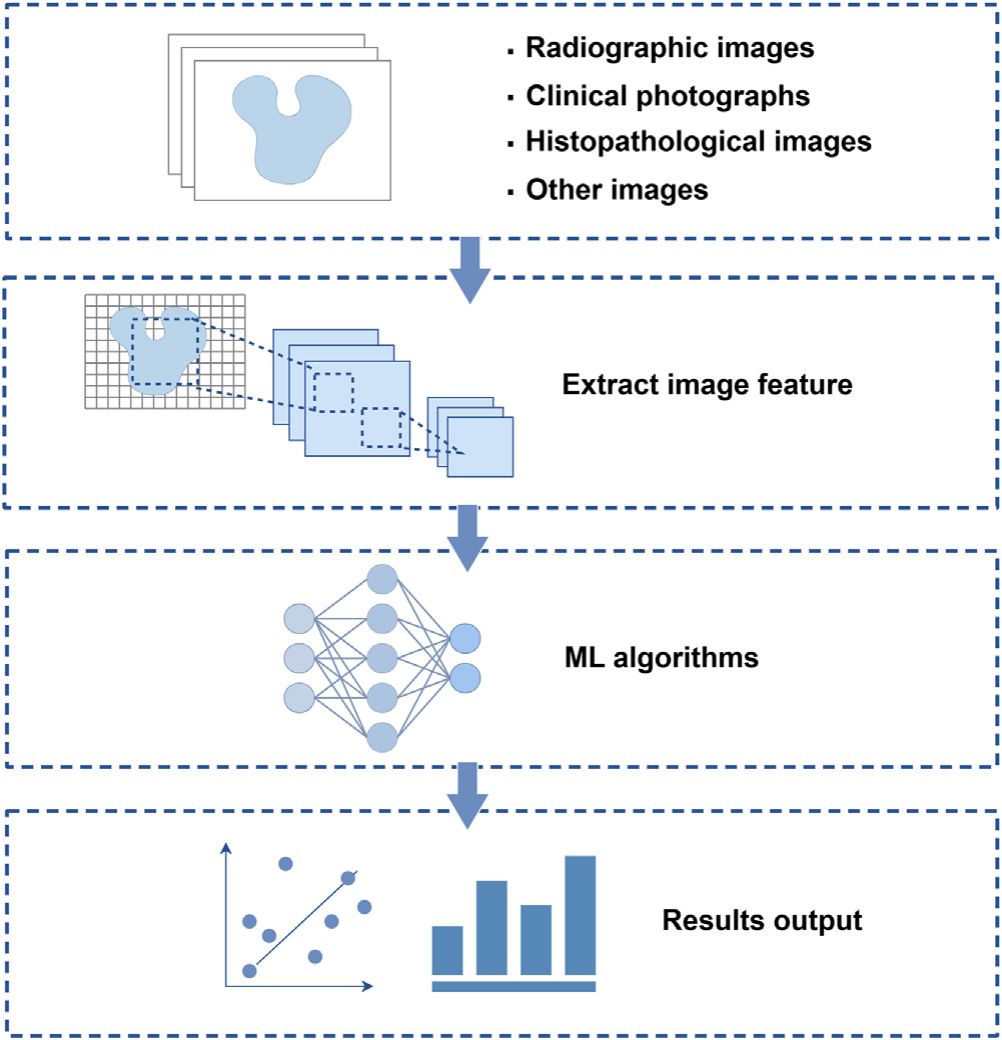
Working principle of AI image recognition and analysis in the diagnosis of oral diseases illustrating the process of extracting feature data from different types of images to train ML/DL algorithms, and finally assisting in the prediction of results.

Image recognition is a fundamental task in computer vision. In dentistry, the screening, diagnosis, and treatment planning of most oral diseases largely rely on the interpretation of image data,^10^ including clinical photographs, radiographs, and histopathological images. DL models trained on a large-scale datasets are instrumental in assisting clinicians in accurate and efficient decision-making. Notably, CNN has emerged as the predominant DL framework for image analysis, demonstrating exceptional performance in image classification and pattern recognition tasks in dentistry. Figure 2 illustrates the simplified working principle of AI image recognition and analysis in the diagnosis of oral diseases.

#### Radiographs

The application of AI in oral imaging includes 2 key aspects: image processing and image analysis. The “as low as reasonably achievable” principle must be strictly followed to minimize radiation exposure during radiography. However, low-dose imaging techniques inevitably introduce challenges such as increased image noise and artifacts, leading to a reduction in image quality and affecting clinical diagnostic accuracy. DL-based image optimization algorithms^11^ and reconstruction techniques^12^ have demonstrated remarkable efficacy in noise reduction and artifact correction for both CT and magnetic resonance imaging (MRI) images. The preliminary diagnostic assistance tasks of AI include segmentation, classification, and labeling of teeth, bones, and other anatomical structures.^13^ AI-powered automated segmentation systems capable of accurately identifying teeth and jaw structures from imaging modalities, including intraoral radiographs,^14^ cone-beam computed tomography (CBCT) images,^15^^,^^16^ and panoramic radiographs,^17^ have recently been developed. These systems have achieved accuracy comparable to that of experienced radiologists while significantly improving operational efficiency.

#### Clinical photographs

Computer vision applications include the analysis of intraoral photographs, usually captured by smartphones and digital cameras. The initial diagnosis of some dental diseases mainly depends on visual examination. Natural-light dental photographs up to a certain quality standard are representative of the human eye-perceived clinical reality. AI recognition of lesion area photographs has received considerable research attention in recent years, and its clinical applications are multifold.

DL-based image recognition can be applied to intraoral photographs of common oral mucosal diseases.^18^^,^^19^ In addition, oral potentially malignant disorders (OPMDs), comprising lesions associated with an increased risk of malignant transformation, have complex clinical features characterized by changes in the color, texture, and consistency of the oral mucosa and skin. Oral photograph-based identification of OPMDs has a key role in oral cancer screening.^20^ Moreover, DL-based oral cancer screening systems expedite the diagnosis and treatment of early-stage oral squamous cell carcinoma (OSCC).^21^^,^^22^ In prosthodontics, a computer vision system is used to perform color matching in tooth restoration. Conventional visual color matching is often influenced by subjective factors. The computer vision color matching significantly reduces interference and improves the accuracy of color matching.^23^ DL-based caries screening systems are used to analyze oral panoramic photographs via cloud-based servers, and have achieved a satisfactory disease recognition effect.^24^, ^25^, ^26^

#### Histopathological images

Histopathologic examination remains the gold standard for the diagnosis of oral cancer and potentially malignant lesions of the oral cavity. Histopathological images are highly standardized medical images, and the task of image diagnosis depends on the expertise and subjective judgment of the clinicians. AI extracts, screens, and analyzes data features of histopathological images, such as edges, colors, shapes, and even more complex characteristics, and integrates imaging and clinical data information. Currently, it is applied in the screening, diagnosis, differential diagnosis, and prognosis prediction of several oral lesions.^27^, ^28^, ^29^

#### Other images

Confocal laser endomicroscopy (CLE) is a high-resolution, real-time, and relatively noninvasive optical microendoscopy technology. The key clinical application of CLE in oral cancer surgery is in the identification and accurate resection of tumor margins.^30^ DL- and AI-assisted CLE may improve diagnostic accuracy.^31^ Hyperspectral Image (HSI), a 3D datacube that combines spatial and spectral information, is another optical technology that promotes noninvasive examination. AI-based HSI has applications in the diagnosis of oral diseases. For example, Wang et al.^32^ used a hybrid 2D/3D CNN model to classify the fluorescence images of tooth samples and reported an accuracy of 96.43% in the detection of early caries. HSI provides reliable tissue diagnosis by capturing the subtle spectral differences of mucosal pathologic tissues. CNN-assisted HSI can distinguish normal and SCC tissues in surgical specimen images,^33^ providing efficient screening and diagnosis of oral cancer and guiding the intraoperative identification of tumor margins.^34^

### NLP in dentistry

The development of NLP began in the early stages of AI research.^35^ Currently, NLP has an auxiliary role in dentistry, considerably improving the efficiency of dental diagnosis and treatment and patient experience. Large language models (LLMs) such as ChatGPT and DeepSeek are well-known examples of DL models in NLP^36^ that are gradually expanding to dentistry.^37^ These involve clinical decision support, documentation, and patient management and contribute to advances in medicine and surgery. Notably, along with exploring their specific application in dentistry, ethical and data security regulatory issues must be addressed to ensure the safe and effective integration of NLP models into large-scale adoption in clinical practice.

In the public health domain, NLP technology-based chatbots and virtual assistants provide personalized oral health advice remotely. Patients description of symptoms or posing of queries in nontechnical language can be analyzed and translated into a structured vocabulary and classification to simplify in-person consultation arrangements. The analyzed and classified results, such as possible disease diagnosis, treatment options, and medical care, can be fed back to the patient.^38^^,^^39^

**Fig. 3:**
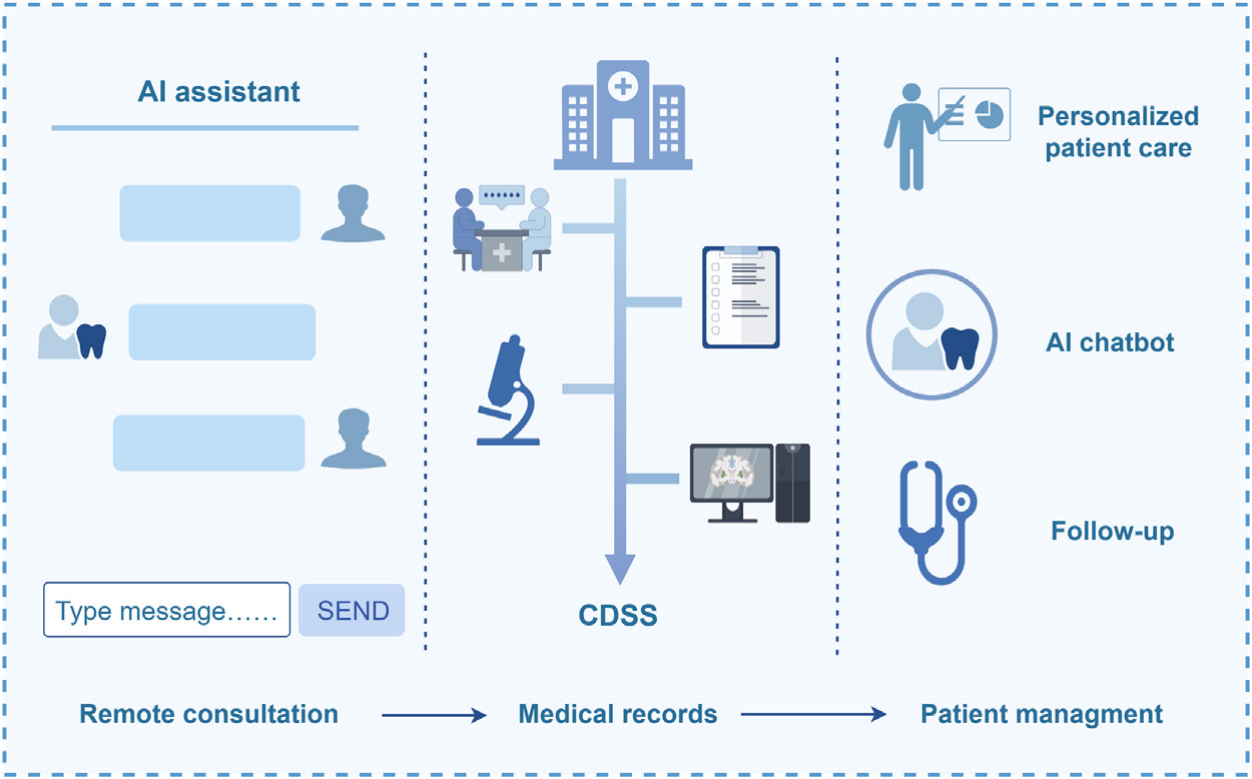
Application scenarios of NLP in dentistry. An illustration of the application logic of AI-based technology in the process workflow of oral healthcare, namely “remote consultation platform–medical records–follow-up management.” EMR, Electronic medical record.

In addition, NLP revolutionizes in-hospital workflows (Fig. 3). EMR systems record patients’ clinical data from the initial consultation. NLP facilitates EMR automation, including speech-to-text conversion and structured data generation. In addition, it helps extract key insights from EMRs, literature, and imaging and pathological diagnosis reports to assist diagnosis and treatment decisions.^40^ Ameli et al.^41^ developed a Clinical Decision Support System (CDSS) using NLP techniques such as Bidirectional Encoder Representations from Transformers (BERT) to predict the stage and grade of periodontitis by analyzing dental charts and clinical records, The system achieved an accuracy of 77% and 75% for stage and grade predictions, respectively, aiding early diagnosis.

Furthermore, extended medical services play a crucial role in healthcare. AI-powered voice-to-text technology has increasingly been adopted for nursing and follow-up management of patients with chronic disease and postoperatively.^42^ This innovation enhances care quality and patient adherence while significantly reducing related costs.

### Behavior recognition technology

Behavior recognition technology involves collecting behavioral data via cameras/sensors and using CNN, GCN, bone point tracking, and temporal modeling (eg, RNN) to identify action patterns and transform behaviors into structured data.^43^ Pose recognition technology (eg, joint motion capture) is used to analyze abnormal behavior characteristics such as falls and shoving, which are often used as intelligent monitoring-based support for wearable equipment. Robots equipped with advanced AI algorithms can significantly improve surgery precision and accuracy in head and neck oncology surgeries. These robots can analyze the patient’s anatomy using advanced sensors and imaging techniques and provide real-time detailed feedback to the surgeon to accurately navigate complex anatomical structures and update surgical plans.^44^ AI-based intraoperative navigation systems can help adjust the trajectories for the robots, facilitating more accuracy in invasive operations. The use of such robotic systems in implant surgery can help dentists achieve optimal implant placement.^45^

## Applications of AI in oral diseases

### Endodontics

In endodontics, disease diagnosis and treatment strategies primarily rely on clinical images. The applications of AI-based algorithms include accurate identification of complex root canal systems and periapical lesions, marking of abnormal tooth structures, and assistance in root canal treatment. The main DL models described in the literature include U-Net, AlexNet, You Only Look Once (YOLO), CNN, GoogleNet, Visual Geometry Group (VGG), Segment Anything Model (SAM), ResNet50, and DetectNet. This section elucidates the main performance indicators of each model: recall, precision, F1 score, sensitivity, specificity, positive predictive value (PPV), negative predictive value (NPV), and area under the curve (AUC).^46^ These indicators aid in quantifying performance and conducting comparative analyses (Table 1).

**Table 1:** AI applications in endodontics.

Applications	Data input	AI Models	Performance	Year	Ref.
Detection of caries	522 bitewing radiographs	YOLO8	Precision: 84.83%, recall: 79.77%; F1-score: 82.22%	2025	Bayati et al.^47^
	380 periapical radiographs	MI-DCNNE	Classification accuracy: 99.13%	2022	Imak et al.^48^
	1000 panoramic radiographs	U-net CNN	Precision: 0.831; recall: 0.826; F1-score: 0.828	2022	Song et al.^55^
Detection of periapical lesions	400 panoramic radiographs	U^2^-net	Precision: 0.82; recall: 0.77; F1-score: 0.8	2024	Boztuna et al.^56^
	Periapical radiographs	AlexNet, GoogleNet, Vgg19, ResNet50	Image recognition accuracy: 88.65-92.91%	2021	Li et al.^57^
	Periapical radiographs	AlexNet, ResNet101, ResNet 50, Google Net	Image recognition accuracy: 87.88-96.21%	2022	Chou et al.^58^
	109 CBCT images	CNN	Detection accuracy: 92.8%	2020	Orhan et al.^59^
	92 panoramic radiographs and CBCT images	CNN	Performance of AI using CBCT images was better than that with panoramic radiographs	2024	Kazimierczak et al.^60^
Root canal treatment	Periapical radiographs	ANN	Accuracy in locating the apical foramen: 93%	2012	Saghiri et al.^61^
	597 periapical radiographs	CNN	Accuracy in image segmentation of root canal fillings: 88%	2024	Çelik et al.^62^
	922 CBCT images	YOLOv5x	MB2 detection model - Sensitivity: 0.92; Precision: 0.83; F1 score: 0.87	2023	Duman et al.^63^
	135 CBCT images	U-Net, residual U-Net, Xception U-Net	C-shaped root canal detection model - Sensitivity: 0.720-0.786; PPVs: 77.6-80.0%	2021	Sherwood et al.^64^

Early diagnosis of dental caries holds much significance. The conventional method involves clinical examination and radiography. In recent years, AI has been used to train and establish a dental caries detection model for diagnostic assistance and improved efficiency. Bayati et al.^47^ reported that their YOLOv8 AI model improved the detection accuracy of proximal caries in intraoral bitewing radiographs (overall accuracy: 84.83%). Imak et al.^48^ reported that their multi-input DCNN ensemble (MI-DCNNE) model was highly successful in diagnosing dental caries based on root tip radiographs (accuracy: 99.13%). AI assistance has significantly improved the accuracy rate of clinicians in diagnosing dental caries on CBCT images.^49^ For early childhood caries (ECC), visual and tactile examination is the preferred diagnostic method. An AI-based model that was used to detect and classify ECC in intraoral photographs reported a detection accuracy of 97.2%.^50^ Furthermore, Mehdizadeh et al.^51^ expanded its application scope, based on the analysis of clinical photograph data, to include wide-scale dental caries screening for the general population, which is expected to effectively promote public dental disease screening. The proportion of older adults with root caries has increased significantly. In a detection of secondary caries, the SVM algorithm achieved an accuracy of 97.1% as well.^52^ These results indicate that the AI-based detection systems are helpful in enhancing the detection ability of caries, thereby improving diagnostic accuracy and clinical effectiveness. However, the model’s performance is influenced by specific factors. Owing to the limitations of 2D imaging, panoramic radiographs are relatively limited in detecting caries on the occlusal, labial, and lingual surfaces of premolars.^53^ Moreover, the assessment results of the depth and location of the caries in the affected tooth was inconsistent with the assessment results of clinicians.

The diagnosis of periapical lesions determines the subsequent treatment strategy. Imaging is the main auxiliary diagnostic method.^54^ Song et al.^55^ and Boztuna et al.^56^ used CNN-based algorithms for periapical lesion segmentation from panoramic radiographs. The CNN model demonstrated a better diagnostic performance than panoramic radiographs. Based on a previous study,^57^ Chuo’s research^58^ focused on improving image segmentation to enhance accuracy. Among the several models, one reached the highest accuracy of 96.21%. Orhan^59^ studied the detection performance of a D-CNN-based system on CBCT images, which achieved a reliability of 92.8% in correctly identifying periapical lesions, similar to that of manual segmentation. A comparative study of the accuracy of image diagnosis of periapical lesions revealed that the detection performance of AI in CBCT images was better than that in panoramic radiographs, with high sensitivity and specificity.^60^ This result is partly due to the better imaging ability of CBCT, ie, providing finer details of dental and bony structures, thus overcoming the limitations of panoramic imaging.

Accurate localization of the anatomical root canal foramen is a critical step in root canal treatment. Radiographic examination and electronic apex locators are the 2 most common techniques for determining apical position. Saghiri et al.^61^ ANN-based model for locating apical foramen demonstrated a more accurate work length. Root canal filling has a significant impact on root canal treatment outcomes. An CNN-based image analysis technology to evaluate the success of a root canal filling has recently been developed.^62^ Postoperative complications of root canal treatment have attracted the attention of dentists. Residual pulpitis and post-treatment periodontitis are partly caused by missing or aberrant root canal systems. DL algorithms for identifying root canal anatomical variations, including the second mesiobuccal (MB2)^63^ and C-shaped root canals,^64^ have reportedly achieved good results.

These findings highlight the significant potential of DL systems in accurate identification of complex root canal morphologies, thereby enhancing the clinical success rate of endodontic treatments while optimizing clinicians’ workflow efficiency. However, application of AI in clinical settings remains in nascency. Manual errors in image labeling will affect the accuracy of the verification results of AI models.^65^ Moreover, the results are dependent on the source and quantity of the training dataset. Resolution of these issues can enhance the clinical utility of AI models in endodontics.

### Periodontics

Periodontitis is a common oral disease. Ongoing research focused on applying AI in periodontitis prevention and treatment, aiming to enhance efficiency in screening, early and auxiliary diagnosis, prognosis prediction, and long-term follow-up monitoring.

At present, the related research on the application of AI in the prevention and treatment of periodontal disease has demonstrated excellent results and promising prospects. Based on the 2017 classification of periodontal diseases,^66^ Ossowska et al.^67^ used ANN to detect alveolar bone loss. The introduction of clinical guidelines for the staging of periodontal and peri-implant diseases necessitated imaging evaluation for diagnosis and treatment planning.^68^ Visual inspection may miss the presence of early periodontitis. Some studies have focused on automated detection and assessment of periodontal bone loss. Danks et al.^69^ used a neural network model to detect dental landmarks on periapical radiographs, objectively calculate periodontal bone loss, and evaluate periodontitis severity stages, reporting that the model performed best for single root teeth. Do et al.^70^ developed and validated an AI-based system using the YOLOv8 for the automated staging and grading of periodontitis from panoramic radiographs and reported that the accuracy of the alveolar bone, cemento-enamel junction, and tooth detection models were >80%. These models are being continuously optimized and improved by combining them with the computer-aided design technology^71^ or integrating two single models.^72^

In addition to image data analysis, AI has been used to assist in noninvasive clinical examinations of periodontal tissues, namely, ultrasonic probing to measure periodontal pocket depth.^73^ Although its accuracy is less than that of manual probing, it may offer a more comfortable visiting experience for patients after optimization and improvement.

In summary, the DL-based models perform exceptionally well in the assessment of bone loss on radiographs and have an immense application potential. However, the current models are limited by its dependency on specific datasets and lack of external validation, hindering its promotion in practical applications. Further model optimization, dataset expansion, and clinical setting-based system verification are needed to support its large-scale promotion and application in the future.

### Oral mucosal diseases

The differential diagnosis of oral mucosal diseases poses a challenge for inexperienced clinicians. For example, oral lichen planus (OLP) manifestations lack specificity, and are similar to that of other oral mucosal diseases, such as oral leukoplakia (OLK), erythema multiforme, and discoid lupus erythematosus, which along with the complex etiologies of these diseases may lead to misdiagnosis.

AI-assisted recognition and analysis of clinical photographs of oral mucosal lesions aids in disease diagnosis. Jurczyszyn et al.^74^ used ANN that incorporated 3 texture features derived from image analysis of the oral mucosa to identify OLK and reported a diagnostic sensitivity and specificity of 100% and 97%, respectively. Keser et al.^75^ developed a DL-based method for identifying OLP using clinical photographs and reported a diagnostic 100% accuracy. Yu et al.^76^ explored the OLP diagnostic potential of different AI platforms using clinical photographs of different anatomical sites and reported the reduced efficiency of these platforms in identifying lesions in uncommon sites and complex cases.

The identification, differentiation, and risk prediction of OPMDs require particular attention. Notably, OSCC commonly develops from the gradual malignant transformation of OPMDs.^77^ This biological continuum results in considerable overlap of clinical manifestations of OSCC and its precursor lesions. Therefore, early detection of OPMDs is crucial for effective oral cancer prevention. DL models have been proved to accurately distinguish OSCC and OPMDs in clinical photos.^78^ Moreover, CNN-based models reportedly achieved 100% and 90%, sensitivity and specificity, respectively, in classification.^79^ The prediction of malignant transformation may help reduce the incidence and mortality rate of oral cancer by facilitating early intervention. Zhang et al.^80^ developed a CNN-based oral mucosa risk stratification model to classify nondysplastic oral mucosa and oral cancer hematoxylin and eosin (H&E) slides. The model successfully identified patients with OLK at high risk of oral cancer development and may assist in the early diagnosis and prevention of oral cancer.

In summary, CNN-based models have been able to analyze clinical photographs and histopathological images of oral mucosal lesions with extremely high accuracy. However, some issues merit consideration. Comparative analysis of existing AI systems for OLP reveals inconsistent diagnostic performance,^81^ primarily because of limited clinical sample size and selection bias, which undermine the reliability and stability of AI platforms in lesion recognition.

Therefore, clinicians should avoid over-reliance on AI outputs and prioritize established diagnostic gold standards. Future research should focus on expanding sample diversity and size to enhance model robustness. With further refinements, AI may become a valuable tool for identifying oral mucosal lesions and assessing malignant transformation risks.

### Oral and maxillofacial surgery

**Fig. 4:**
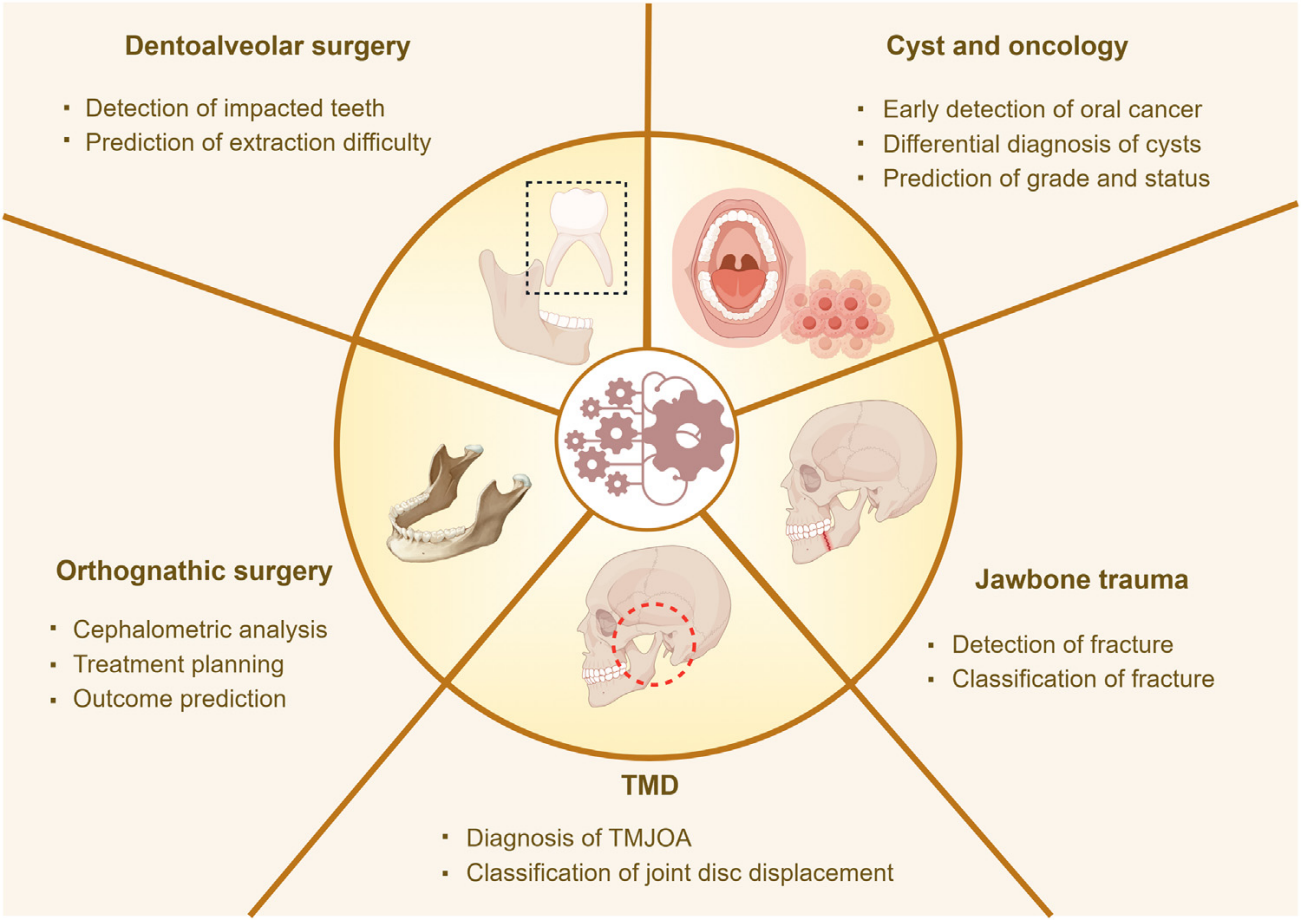
Applications of AI in oral and maxillofacial surgery. TMJOA, Temporomandibular joint osteoarthritis.

The applications of AI in oral and maxillofacial surgery have significantly expanded across various diagnostic and therapeutic domains, including the interpretation and analysis of image data, preoperative assessment of tooth extraction, differentiation of cystic lesions, prediction and diagnosis of tumors, minimally invasive surgical assistance, personalized surgical plans, intraoperative navigation, postoperative follow-up, and patient management.^82^ This section has explored the integration and application of AI technology in oral surgery. Figure 4 provides an overview of the applications of AI in oral and maxillofacial surgery.

#### Dentoalveolar surgery

Tooth extraction remains a common procedure in oral and maxillofacial surgery. Currently, panoramic radiographs or CBCT images play a crucial role in the detection of impacted teeth and the assessment of tooth extraction difficulty. AI image recognition applications have proved their effectiveness in detecting impacted teeth and analyzing their spatial relationships with adjacent anatomical structures.

Kuwada et al.^83^ compared the performance of 3 DL systems for classifying maxillary impacted supernumerary teeth, one of which had the highest diagnostic accuracy of 96%. Kucuk et al.^84^ YOLO and RT-DETR hybrid model significantly reportedly improved diagnostic accuracy and efficiency. Akdoğan et al.^85^ developed a simple AI-assisted tool for assessing the surgical difficulty of impacted mandibular third molars using radiographs and reported an accuracy of 97 %. However, most studies have focused on radiograph analysis. Kwon et al.^86^ comprehensively considered radiographs and clinical factors that influence surgery (patient sex, age, and weight and surgeon’s operation skills) to develop a concatenated model combining a CNN and a multi-layer perceptron to more accurately predict the extraction time of the mandibular third molar.

Most studies on the detection of impacted teeth have involved panoramic radiographs; however, the 3D structure of the impacted teeth need consideration in clinical practice. Hence, it is necessary to perform these assessments on 3D images. AI was reported to have a high accuracy in the detection of impacted third molars and the number of root canals and their adjacent structures (inferior alveolar canal and maxillary sinus).^87^

Notably, the efficacy of these applications fundamentally depends on 2 critical capabilities: precise localization of maxillofacial anatomical landmarks and accurate segmentation of the teeth, jaws, and soft tissue.^88^^,^^89^ Moreover, previous studies have reported that model performance improvement can be achieved by model fusion to integrate the advantages and reduce the limitations of each model. In clinical application, several factors need to be considered comprehensively for analysis and judgment.

#### Head and neck cyst and tumors

Cysts and tumors represent 2 principal disease categories in oral surgical practice. This section systematically explores the applications of AI models in head and neck cyst and cancer. Table 2 presents the comparative analysis.

**Table 2:** AI applications in head and neck oncology.

Applications	Data input	AI models	Performance	Year	Ref.
Detection of cystic lesions	150 CBCT images	GoogLeNet, Inception-v3	Accuracy, sensitivity, and precision: >90%	2020	Lee et al.^90^
Diagnosis of OKC	2157 histopathological images	SVM, RF, XGboost	Integration of pathological information from multiple pathological slides by the model demonstrated a greater advantage than that of a single slide	2024	Cai et al.^91^
Differential diagnosis of OKC	350 CBCT images	CNN	Sensitivity: 87.2%; specificity: 82.1%; accuracy: 84.6%; and F1 score: 85.0%	2022	Chai et al.^92^
Image segmentation of ameloblastoma	79 CT images	Mask R-CNN	DICE index*: 0.874	2024	Xu et al.^95^
Classification and detection of oral cancer	700 clinical photographs	DenseNet121 and R-CNN	DenseNet121 classification accuracy: 99% and R-CNN detection accuracy: 76.67%	2022	Warin et al.^97^
Prediction of OSCC grade and lymph nodal status	40 patients	J48, MLP, NB, and KNN	Prediction accuracy: >90%	2020	Romeo et al.^99^
Detection of OSCC	1224 histopathological images	GoogLeNet, ResNet101, VGG16, XGBoost	Diagnostic accuracy: 99.3% and sensitivity: 98.2%.	2023	Ahmed et al.^104^
	90059 images	EfficientNet B0	Algorithm can train the AI to assess the cellular and structural atypia of OSCC, and is applicable for the diagnosis of OSCC	2022	Oya et al.^98^
Classification of enlarged cervical lymph nodes	276 CT images	CNN	Overall accuracy of 87.50%, which was higher than that of radiologists	2022	Zhang et al.^100^
Identifying lymph node metastases	89 patients	CNN	Sensitivity: 65%; Specificity: 68%	2024	Esce et al.^101^
Tumor margin assessment	2194 HSI	Linear SVM, ResNet	Performance in evaluating the tumor margins is comparable to the traditional intraoperative tumor margin assessment	2022	Pertzborn et al.^102^

⁎ DICE index is a widely used evaluation metric in medical image segmentation.

The differential diagnosis of odontogenic cysts poses challenges for inexperienced clinicians. Reliable diagnosis is contingent upon supporting evidence including clinical manifestations and pathological and imaging examinations. Therefore, research on the clinical application of DL in common jawbone lesion management holds much significance. Lee et al.^90^ developed a DL model to detect and classify odontogenic lesions (odontogenic keratocysts [OKC], dentigerous cysts, and periapical cysts) in CBCT and panoramic images and reported a sensitivity and specificity of >77% each, with the performance in CBCT images being significantly better than that in panoramic radiographs. Among odontogenic cysts, OKC are more prone to recurrence postsurgery. Cai et al.^91^ DL algorithm-based diagnostic and prognostic model for OKC using H&E images demonstrated a promising performance in identifying OKC. Some cystic lesions, such as OKC and ameloblastoma, have similar imaging manifestations albeit completely different treatment principles and prognoses. Chai et al.^92^ used a CNN-based model to differentiate ameloblastoma from OKC using CBCT images, which demonstrated superior discriminative diagnostic accuracy compared with clinicians. Although the model performed well, it lacked external validation and rich data sets; therefore, future studies should focus on strengthening the generalization and credibility of the model.

In addition, AI has been used in oral cancer identification and risk prediction.^93^ Clinical images are reliable data for the initial diagnosis of tumors. Ameloblastoma have local invasiveness and the possibility of recurrence,^94^ which have always garnered attention among clinicians. Xu et al.^95^ trained a Mask R-CNN model to segment ameloblastoma using CT images. Pathological examination is the gold standard for tumor diagnosis. A study on the pathological diagnosis of ameloblastoma and ameloblastic carcinoma has demonstrated the usefulness of AI-based diagnostic models.^96^ Warin et al.^97^ used CNN-based models to classify and detect OSCC from photographs, which achieved excellent performance. Such trained models can potentially be applied to automate classification for oral cancer screening. The new training method of Oya et al.,^98^ that considered cell and structural atypia, aimed to improve the accuracy of CNN-based algorithms in SCC phenotypic diagnosis to facilitate the development of more reliable, objective, and accurate diagnostic tools.

Tumor staging and grading strongly affect clinical decision-making and prognosis evaluation. Romeo et al.^99^ applied radiomics to predict the tumor grade and lymph node status of OSCC. Zhang et al.^100^ developed a model for classifying and detecting enlarged cervical lymph nodes in CT images to distinguish benign lymphadenopathy, metastatic lymph nodes, and lymphoma and reported a significantly higher classification accuracy than that of radiologists (0.875 vs 0.627).

Furthermore, Esce et al.^101^ used CNN to predict lymph node metastasis in primary tongue SCC from histopathological images, with the top-performing model achieving 65% and 86% sensitivity and specificity, respectively.

Accurate assessment of surgical margins is critical for determining complete resection of head and neck tumors. HSI and ML combination model of Pertzborn et al.^102^ for margin evaluation, aimed at overcoming the limitations of intraoperative biopsies and demonstrated comparable effectiveness to conventional biopsy albeit with greater speed and accuracy.

Additionally, the integration of molecular biology with ML has advanced head and neck tumor research. Wang et al.^103^ developed a macrophage-related signature (MRS) model using ML and weighted gene co-expression network analysis to identify relevant genes in SCC, which demonstrated its potential in prognostic evaluation and mechanistic exploration.

#### Temporomandibular disorders

Temporomandibular disorders (TMD) comprise a spectrum of conditions, characterized by multifactorial etiopathogenesis and heterogeneous clinical presentations. Recent advances in AI hold much promise in mitigating these issues, thereby improving the efficiency and accuracy of TMD diagnosis.

Temporomandibular joint osteoarthritis (TMJOA) is a major subtype of TMD. Currently, CT is the recognized reference standard for TMJOA diagnosis,^105^ and a CBCT image-based AI diagnostic tool has recently been reported.^106^^,^^107^ However, panoramic radiographs are the most common method for initial TMJOA screening. Choi et al.^108^ developed a DL-based tool that detects osteoarthritis in panoramic radiographs, enabling TMJOA diagnosis in facilities without CT. MRI is the standard modality for diagnosing TMJ disc displacement. Orhan et al.^87^ employed ML models, including k-nearest neighbors (KNN) and random forest (RF), to classify condylar changes and disc dislocations in MR images. DL-trained AI models have been used to assist clinicians in the diagnosis of disc displacement and reduction. AI-assisted strategies have been reported to significantly improve the diagnostic accuracy of MRI for anterior TMJ disc displacement.^109^ Lasek et al.^110^ used AI technology to automate the measurement of TMJ space width in ultrasound images. The model demonstrated a high accuracy and is expected to become a reliable, noninvasive diagnostic tool.

In summary, AI can provide faster and more accurate diagnosis of TMD by using data from imaging modalities such as MRI, CBCT, and ultrasound. Emerging research is using the more easily available clinical data with the assistance of AI prediction models for the early detection of TMD.^111^ Importantly, researchers should extensively refine and comprehensively evaluate AI models before their application in clinical practice.

#### Jawbone trauma

Jawbone fractures are common cases in oral and maxillofacial surgery. Current research primarily focuses on fracture diagnosis and surgical design, using DL-based models for tasks such as detecting fracture sites and classifying fracture types.

Warin et al.^112^ used the DenseNet-169 and ResNet-50 CNN algorithms to detect mandibular fractures in panoramic radiographs. The models performed well, with a detection accuracy of 90%, exceeding expert-level performance. A similar study on a model using spiral CT data achieved a segmentation accuracy of >90%.^113^ A comparative study evaluated the performance of DL models in detecting the types of mandibular fractures. Yari et al.^114^ trained the YOLOv5 algorithm and detected mandibular fracture types based on 6 anatomical locations: symphysis, body, angle, ramus, condylar neck, and condylar head. The results revealed that the accuracy was highest in body (96.21%) and joint (95.87%), and lowest in angle fractures, demonstrating that DL with CNN may assist in the diagnosis of condylar fractures. Considering that the trained models often lack external validity for diagnosis, one study collected panoramic radiographs from 2 hospitals with and without condylar fractures. The model demonstrated low performance for datasets from different hospitals, whereas the combined dataset demonstrated high performance, indicating the high reliability and accuracy of CNN in mandibular fracture diagnosis. Furthermore, AI has been applied in assisting clinicians to locate nasal bone fractures on CT images.^115^

#### Orthognathic surgery

AI has been used for cephalometric analysis, facial contour assessment, surgical planning, and treatment outcome prediction in orthognathic surgery. Khosravi-Kamrani et al.^116^ established a statistical prediction model for treating patients with Class III malocclusion that analyzed the cephalometric measurement data and classified the patients for providing personalized treatment plans. The results revealed that patients with mandibular protrusion most frequently underwent orthognathic surgery. Lin et al.^117^ used the ML-based XG-Boost algorithm to determine the cephalometric measurement indicators that could predict whether patients with unilateral cleft lip and palate would need orthognathic surgery in the future. The model predicted that such patients would need surgery to correct the sagittal bony difference at the age of 6 years, with an accuracy of 87.4%.

In addition, in-depth research on AI-based soft tissue change prediction has been conducted. Current DL models can predict 3D postoperative soft tissue contours with clinically acceptable accuracy. Seo et al.^118^ used AI to evaluate the soft tissue changes in patients with cleft lip after bimaxillary surgery. Lin et al.^119^ developed a CNN model based on 3D features, using the transfer learning method to quantitatively assess facial symmetry and intervention outcomes, demonstrating significant improvement after orthognathic surgery.

In summary, AI-based models demonstrated excellent performance in the detection of impacted teeth, the detection and differentiation of jawbone cysts/tumors, TMDs, and orthognathic surgery analysis. The technology has been applied to other common conditions such as salivary gland diseases^120^ and maxillary sinusitis^121^ as well.

Although AI is gradually transforming conventional diagnosis and treatment, current research remains limited by dataset quality and disease diversity. Improved datasets may yield results better suited to clinical needs.

### Orthodontics

AI has been integrated into advanced orthodontics in diagnosis, treatment planning, and outcome evaluation.

Cephalometric analysis, especially the positioning of anatomical landmarks, is essential in orthodontic diagnosis. Manual positioning is time-consuming and error-prone. Ongoing research is focused on AI-based cephalometric analysis for automated identification of anatomical landmarks.^122^^,^^123^ Image recognition models have been used to analyze cephalometric landmarks via lateral^124^ and anterior-posterior cephalometric radiographs.^125^ Compared with manual recognition, these models demonstrated excellent accuracy and reliability, enabling efficient cephalometric analysis. However, human supervision and adjustment of the automated procedures can further improve the accuracy and efficiency of the AI-based analysis.^126^

Skeletal age assessment is an additional application of AI in orthodontics. As a key indicator of growth and development‌, skeletal maturity ‌has a significant impact on‌ orthodontic outcomes.^127^ Skeletal age can be assessed using wrist or cervical vertebral maturation (CVM) radiographs. Seo et al.^128^ compared 6 DL models for evaluating CVM using lateral cephalometric radiographs and reported an accuracy of >90% for all models. Additional studies have demonstrated the diagnostic accuracy of AI in assessing skeletal age using wrist and CVM radiographs as well.^129^, ^130^, ^131^

Tooth extraction decision-making, a critical component in orthodontic treatment planning, is affected by factors such as patient expectations and clinician experience. AI tools have recently been introduced into the tooth extraction decision-making process.^132^ Ryu et al.^133^ successfully established an AI diagnostic model for tooth extraction based on intraoral photographic image data, classified the degree of crowding by calculating the difference in dental arch length, and analyzed the model’s accuracy. The model demonstrated promising performance in the classification of crowding degree and diagnosis of tooth extraction, which can assist clinicians in orthodontic treatment planning.

Furthermore, AI has been used to predict treatment effect, such as facial soft and hard tissue morphology. An AI system for predicting post-treatment facial morphology has been proven to be clinically acceptable.^134^ Park et al.^135^ developed an automated system to predict teeth, bone, and soft tissue changes after nonextraction treatment for Class II malocclusion. The model demonstrated exceptional predictive accuracy. Moreover, AI systems with a high accuracy in predicting the facial symmetry of patients undergoing orthognathic surgery have been reported.^136^

Although the evolution of AI algorithms enhances the visualization of efficacy prediction and the optimization of decision making, the contribution of human factors to treatment outcomes and the representativeness and reliability of available data merit serious considerations.

### Prosthodontics

AI applications in oral restoration include dental crown design, restoration longevity prediction, and color matching (Table 3). CAD/CAM systems are widely used in dental restoration. The combination of dental software systems and intraoral scanners enables the creation of a 3D surface model of the remaining teeth, based on which, dental restorations are designed and fabricated.^137^

**Table 3:** AI applications in prosthodontics.

Applications	Data input	AI models	Performance	Year	Ref.
Dental crown design and generation	600 digital casts	3D-DCGAN	AI design and natural teeth had the lowest discrepancy in morphology	2023	Ding et al.^138^
	45 dental models	PrintIn DentDesign software	AI-based crown fabrication is more efficient than digital and conventional wax-up fabrication	2024	Liu et al.^139^
	120 tooth preparations using intraoral scans	3D-CNN	Validation accuracy: 60%; sensitivity: 1.00; and precision: 0.50-0.83	2023	Farook et al.^141^
Color matching	580 tooth photographs	RF, KNN, and SVM	Accuracy was 97.93% in the classification of 29 colour values	2025	Karcioglu et al.^144^
Tooth preparation	182 jaw with tooth abutment scans	CNN	Hybrid method achieved accurate extraction of the finish line	2024	Choi et al.^143^
RPDs design	1184 dental arch photographs	CNN	Diagnostic accuracy: >99%; precision: 0.25; recall: 1.0; and F-score: 0.4	2021	Takahashi et al.^146^

Several studies have evaluated the feasibility and accuracy of AI models in dental crown designing. A 3D-DCGAN-trained ML model for designing dental crowns has been reported.^138^ The personalized crown’s shape could simulate the morphology and satisfactory mechanical properties of the natural tooth. In addition, AI-based crown designing is more time efficient than other design methods^139^^,^^140^ Another study used a 3D-CNN model to generate partial crowns based on intraoral scan data.^141^

Accurate determination of the tooth preparation finish line affects the marginal fit of the restoration. However, conventional methods are often difficult, time-consuming, and complex. CNN-based models have recently been used to determine the finish line.^142^ The model of Choi et al.^143^ which combined DL with CAD, achieved reliable and precise detection of the finish line.

Color matching is an important procedure in restoration; however, its accuracy is influenced by subjective factors. Kim et al.^23^ developed a digital color-matching device for determining tooth color using the SVM algorithm and reported an average matching accuracy of >90% for all devices, proving its utility for high-precision quantitative measurement of tooth color. The study by Karcioglu et al.^144^ further overcame the influence of light source condition, reporting more accurate results.

The design of removable partial dentures (RPDs) exhibits significant individual differences. AI-based models can help offer a personalized design plan for RPDs to improve the accuracy of dental restoration.^145^ The preliminary classification of the dental arch types is the first step in the automated design of RPDs. A CNN-based model was reported to have achieved a classification accuracy of >99%.^146^ Nevertheless, further clinical studies are required to validate the precision of AI systems in designing RPDs.

Further advances in DL can promote the intelligentization of restorative dentistry, including the design of inlays, full crowns, and RPDs and color matching, as well as the quality assessment of the final restoration products,^147^ significantly improving the time-consuming and labor-intensive production processes.

Current research on AI applications in prosthodontics remains in a nascent stage, and most are focused on common restorations,^147^ and sufficient clinical trials for evaluation are lacking.^148^ Future studies on AI-driven restorations should focus on design precision, balancing aesthetic restoration and functional reconstruction and achieving a more efficient workflow.

## Challenges and future perspectives

AI integration into dentistry is a critically important topic; however, its application in clinical settings is challenged by data availability, algorithmic limitation, and data privacy issues.

The performance of AI algorithms is dependent on the quality and representativeness of the training dataset. At the data acquisition level, local data storage limitations and clinical information sensitivity hinder effective data collection and utilization. Furthermore, data errors and biases, imbalanced sample selection, manual annotation inconsistencies, and data collection equipment variations may be inadvertently learned and perpetuated by the AI model, leading to poor performance when applied to other datasets.^149^

Data privacy and security represent another major challenge.^150^ Although LLMs have demonstrated considerable potential in clinical decision support, medical education, and research acceleration,^151^ open data access and security vulnerabilities may expose sensitive user information, leading to significant data security risks. The AI algorithms driving these models often lack transparency in data interpretation. Moreover, the deployment of LLMs in clinical settings requires careful consideration of ethical issues, such as patient privacy, informed consent, and legal implications.

Several strategies have been proposed to address these challenges.^152^ The improved data interoperability and sharing may contribute to dataset standardization. Representational biases can be mitigated by establishing data standards and intentionally using diverse and inclusive datasets, rendering clinical outcomes more equitable and reliable. Ethical challenges require clear regulations and guidelines. Policymakers should prioritize patient data privacy and security, seek informed consent, and ensure transparency in data usage. Furthermore, stringent review and monitoring mechanisms should be implemented throughout the AI application lifecycle.

## Conclusion

This review systematically describes the technical pathways and application status of AI in various dentistry specialties. With the continuous breakthrough and evolution of technology, the performance of most of the reported models can match or even exceed dentist-level accuracy, demonstrating immense application potential. However, the clinical applications of AI models in dentistry currently faces dataset dependence, insufficient validation, and data privacy challenges. Future research should focus on promoting the integration of AI in clinical practice via algorithm optimization, multi-center data fusion, and a standardized evaluation system, aiming to develop a more intelligent healthcare ecosystem.

## Author Contribution

**Ying Yuan**: Writing–review & editing, Writing–original draft, Methodology, Investigation, Formal analysis, Data curation, Conceptualization. **Hou Zhou**: Writing–review & editing, Formal analysis. **Bo Wang**: Writing–review & editing. **Chengrun Li**: Writing–review & editing. **Baijun Gong**: Resources; Writing–review & editing, Methodology, Investigation, Supervision. **Zhimin Li**: Writing–review & editing, Methodology, Supervision, Investigation, Conceptualization.

## Conflicts of interest

None disclosed.
